# Bacterial brain abscess in patients with nasopharyngeal carcinoma following radiotherapy: microbiology, clinical features and therapeutic outcomes

**DOI:** 10.1186/1471-2334-12-204

**Published:** 2012-09-03

**Authors:** Peng-Hsiang Fang, Wei-Che Lin, Nai-Wen Tsai, Wen-Neng Chang, Chi-Ren Huang, Hsueh-Wen Chang, Tai-Lin Huang, Hsin-Ching Lin, Yu-Jun Lin, Ben-Chung Cheng, Ben Yu-Jih Su, Chia-Te Kung, Hung-Chen Wang, Cheng-Hsien Lu

**Affiliations:** 1 Department of Neurosurgery, Kaohsiung Chang Gung Memorial Hospital, Chang Gung University College of Medicine, Kaohsiung, Taiwan; 2 Departments of Radiology, Kaohsiung Chang Gung Memorial Hospital, Chang Gung University College of Medicine, Kaohsiung, Taiwan; 3 Departments of Neurology, Kaohsiung Chang Gung Memorial Hospital, Chang Gung University College of Medicine, Kaohsiung, Taiwan, 123, Ta Pei Road, Niao Sung Hsiang, Kaohsiung Hsien, 833, Taiwan; 4 Department of Biological Science, National Sun Yat-Sen University, Kaohsiung, Taiwan; 5 Departments of Medicine, Kaohsiung Chang Gung Memorial Hospital, Chang Gung University College of Medicine, Kaohsiung, Taiwan; 6 Departments of Otorhinolaryngology, Kaohsiung Chang Gung Memorial Hospital, Chang Gung University College of Medicine, Kaohsiung, Taiwan; 7 Departments of Emergency Medicine, Kaohsiung Chang Gung Memorial Hospital, Chang Gung University College of Medicine, Kaohsiung, Taiwan

**Keywords:** Bacterial brain abscess, Nasopharyngeal carcinoma, Therapeutic outcome

## Abstract

**Background:**

This study aimed to analyze the clinical features, causative pathogens, neuro-imaging findings, and therapeutic outcomes of bacterial brain abscess in patients with nasopharyngeal carcinoma (NPC) following radiotherapy.

**Methods:**

NPC patients with bacterial brain abscess were evaluated. Their clinical data were collected over a 22-year period. For comparison, the clinical features, causative pathogens, neuro-imaging findings, and therapeutic outcomes between NPC and non-NPC patients were analyzed.

**Results:**

NPC accounted for 5.7% (12/210) of the predisposing factors, with Viridans streptococci and *Staphylococcus aureus* as the two most common causative pathogens. Significant statistical analysis between the two groups (NPC and non-NPC patients) included chronic otitis media (COM) as the underlying disease, post-radiation necrosis by neuro-imaging, and the temporal lobe as the most common site of brain abscesses. The fatality rate in patients with and without NPC was 16.7% and 20.7%, respectively.

**Conclusions:**

NPC patients with bacterial brain abscess frequently have COM as the underlying disease. Neuro-imaging often reveals both post-radiation necrosis and the temporal lobe as the most common site of brain abscesses, the diagnosis of which is not always a straightforward process. Radiation necrosis can mimic brain abscess on neuro-imaging and pose significant diagnostic challenges. Early diagnosis and treatment is essential for survival.

## Background

Nasopharyngeal carcinoma (NPC) is a rare disease in most parts of the world but is one of the leading causes of cancer among the Chinese, particularly those in southeastern China and Taiwan [1]. Large nasopharyngeal tumors usually destroy the skull base and break down the blood–brain barrier of the central nervous system (CNS). They also often block the nasal airway and Eustachian tube, which may result in rhino-sinusitis or otitis media with or without effusion. To date, clinical research regarding bacterial brain abscesses in NPC patients are limited to diverse bacterial brain abscesses [2,3] or case report study [4], and have less strict selection criteria that include various types of CNS infections or as late sequelae of radiotherapy for NPC [5-8].

The risks of CNS infection are increased in post-irradiation NPC patients with impaired CNS barrier caused by the high prevalence of chronic otitis media [9]. Due to possible benefits of therapeutic intervention, there is a need for better delineation of potential risk factors and clinical features in this specific group of patients.

A hospital-based study provides accurate information about localization of brain abscess, predisposing factors, clinical features, prevalence rate of implicated bacterial pathogens, and causes of fatality. The present study describes therapeutic experiences and attempts to analyze the clinical features, neuro-imaging findings, clinical scores, and measurements to determine the therapeutic outcomes in this special group of patients.

## Materials and methods

### Study population

Microbiological records for abscesses and blood cultures, medical records, and neuro-imaging findings using pre-existing standardized evaluation forms were retrospectively reviewed for patients with bacterial brain abscesses admitted to Kaohsiung Chang Gung Memorial Hospital, a 2482-bed acute-care teaching hospital providing primary and tertiary referral care, in the period 1986–2007.

### Diagnostic criteria of bacterial brain abscess

The diagnostic criteria of bacterial brain abscess were: 1) characteristic computerized tomography (CT) and/or magnetic resonance imaging (MRI) findings; 2) evidence of brain abscess seen during surgery or histopathologic examination; and 3) classical clinical manifestations such as headache, fever, localized neurological signs and/or consciousness disturbance [10,11]. Brain abscesses were defined as nosocomial according to the 1988 guidelines of the Centers for Disease Control [12]. Brain abscesses related to head trauma with skull fracture or neurosurgical procedures were classified as a post-neurosurgical form. Otherwise, patients who presented with no distinctive characteristics or those who had not undergone invasive procedures were classified as the spontaneous form.

### Diagnostic criteria and therapeutic regimens of nasopharyngeal carcinoma (NPC)

In all patients, experienced pathologists diagnosed NPC histologically, while multi-disciplinary teams administered treatment. The hospital’s therapeutic protocol was according to the National Comprehensive Cancer Networks (NCCN) Clinical Practice Guidelines in Oncology-Head and Neck Cancers (USA) and the Kaohsiung CGMH Head and Neck Oncology Group of Chang Gung Memorial Hospital Cancer Center. The therapeutic strategies were as follows: patients with stage I-IIA (AJCC system) were given radiotherapy alone whereas those with stage IIB-IV were given concurrent chemo-radiotherapy (CCRT).

### Exclusion and inclusion criteria

Patients with evidence of brain abscesses not due to bacterial pathogens were excluded from this study. Patients who were initially treated in other hospitals but subsequently transferred to the study hospital for further therapy were included in this study, with the initial clinical data collected in those hospitals used for analysis.

### Clinical assessment

The Glasgow coma scale (GCS) score was determined by neurosurgeons or neurologists as the patient arrived at the emergency room. All of the patients received brain CT scans shortly after arriving at the emergency room. Follow-up brain CT and/or MRI studies were performed for any clinical deterioration, including acute onset of focal neurologic deficits, seizures or status epilepticus, and progressively disturbed consciousness, as well as post-neurosurgical procedures. Hydrocephalus was judged retrospectively by a dilated temporal horn of the ventricle without obvious brain atrophy and/or an Evan’s ratio (the ratio of the ventricular width of the bilateral frontal horn to the maximum bi-parietal diameter) >0.3 on initial CT scans.

The volumes of brain abscesses on admission CT scans were measured. An experienced radiologist who was blinded to the patients’ clinical and biochemical data analyzed the CT scans for volumetric measurements of brain abscess volumes. All images were processed using the imaging processing software (Vitrea version 3.9.0.1, Vital images, Minnesota, U.S.A.) running on an off-line workstation. The volumes were calculated using a semi-automated process. The examiner manually drew regions-of-interest (ROI) in each slice throughout the brain abscess. Contiguous voxels were automatically summed to yield a brain abscess volume. The observer drew the brain abscess twice, at an interval of one month.

A trained research assistant performed the measurements again. Maps of the ROI used for measurement were stored and confirmed by a neurosurgeon. Intra- and inter-observer reproducibility of these measurements was evaluated using intra-class correlation coefficients. For brain abscess volume measurements, the intra-observer agreement was r = 0.99, while the inter-observer agreement was r = 0.99. The “volumes of brain abscesses” indicated the sum of all volumes of brain abscesses if at least two were found.

### Therapeutic regimens

Combined surgical intervention and antibiotic therapy were the mainstays of treatment of bacterial brain abscesses. In the study hospital, surgical management consisted of imaging-guided stereotactic aspiration or craniotomy with complete excision. The contents of the abscess were aspirated using a ventricular catheter via burr hole or through a small craniotomy, which left the capsule alone. Craniotomy and resection of the abscess were defined as excision. The choice of one procedure over another was based on the patient’s age and neurologic condition, the location, stage and type of abscess, and the presence of multiple lesions.

Stereotactic aspiration was the simplest and safest method of obtaining pus for culture. It allowed for the precise localization and decompression of the abscess cavity using a minimally invasive technique, and was valuable in treating deep-seated lesions, lesions in eloquent areas, and multiple abscesses [13]. The combination of third-generation cephalosporins and metronidazole for 8–12 weeks was the mainstay of initial empiric antimicrobial treatment for bacterial brain abscesses. The choice of final antibiotics was guided by the final culture results.

All of the materials from cerebrospinal fluid and/or blood, and/or drainage from the ear or sinuses were cultured for aerobic and anaerobic bacteria, *Mycobacterium*, and fungi. Antibiotic susceptibility was determined using the Kirby-Bauer disc diffusion method (Mueller-Hinton II agars; Becton Dickinson Microbiology Systems, Cockeysville, MD).

### Outcome assessment

Evaluation of therapeutic outcome after discharge and a minimum 18-month follow-up was based on the Glasgow outcome score (GOS) as follows: good recovery, moderate disability, severe disability, persistent vegetative state, and death [14].

### Statistical analysis

Two separate statistical analyses were performed. First, the demographic data between the NPC and non-NPC groups were compared. Categorical variables were compared using the chi-square test or Fisher’s exact test, as appropriate. Continuous variables within the two groups were compared using the independent t-test for parametric data and the Mann–Whitney U test for non-parametric data. Second, significant variables (*p* < 0.05) associated with an NPC patient group were entered into a forward stepwise logistic-regression analysis model that allowed for simultaneous control of multiple factors. Variables with a zero cell count in a 2-by-2 table were eliminated from logistic analysis, while only variables with a strong association with fatality rate (*p* < 0.05) were included in the final model. All statistical tests were two-tailed and were conducted using the SAS software package, version 13.0 (2002, SAS Statistical Institute, Cary, North Carolina).

## Results

### Baseline data of study patients

The 210 patients with bacterial brain abscesses included 156 males (mean age, 46.1 years) and 54 females (mean age, 45.5 years). Bacterial brain abscesses in NPC patients following radiotherapy occurred in 12 of the 210 patients, including 8 with community-acquired infections and 4 diagnosed with nosocomial infections. Aside from NPC, other associated underlying conditions of the 12 patients were listed in Table 1. The characteristics of the 210 patients with bacterial brain abscesses with or without NPC were listed in Table 1.

**Table 1:** Clinical comparisons of patients with bacterial brain abscesses without or without NPC

	***Non-NPC patients***	***NPC patients***	***p value***	***OR***	***95% CI***
	*n = 198 (%)*	*n = 12 (%)*			
*Mean age, years*	*45.8±18.2*	*48.3±12.7*			
*Sex (male/female)*	*147/51*	*9/3*	*1.0*	*1.041*	*0.27-3.99*
*Median (IQR) GCS on presentation*	*15 (14, 15)*	*13.5 (10.3, 15.0)*	*0.39*		
*Underlying diseases*					
* Congenital heart diseases*	*2 (0.1)*	*0*	*1.0*	*0.942*	*0.911-0.975*
* Diabetes mellitus*	*42 (21.2)*	*1 (8.3)*	*0.466*	*0.34*	*0.042-2.69*
* Alcoholism/liver cirrhosis*	*9 (4.5)*	*0*	*1.0*	*0.94*	*0.91-0.97*
* Chronic otitis media*	*20 (10.1)*	*8 (66.6)*	*<0.0001*	*17.8*	*4.9-64.4*
* Postneurosurgical state*	*43 (21.7)*	*5 (41.6)*	*0.151*	*2.58*	*0.778-8.52*
*Neuroimaging findings at presentation*					
*Multiple/ Solitary*					
* Multiloculated*	*41 (20.7)*	*3 (25)*	*0.718*	*1.276*	*0.331-4.929*
* Involved temporal lobe*	*65 (32.8)*	*8 (66.6)*	*0.026*	*4.092*	*1.19-14.1*
* Hydrocephalus*	*32 (16.2)*	*3 (25)*	*0.426*	*1.729*	*0.444-6.74*
* Post-radiation necrosis*	*0 (0)*	*3 (25)*	*<0.001*	*0.044*	*0.023-0.083*
*Clinical features following brain abscess*					
* Fever/chills*	*119 (60.1)*	*10 (83.3)*	*0.135*	*3.319*	*0.708-15.55*
* Headache*	*108 (54.5)*	*9 (75)*	*0.234*	*2.5*	*0.657-9.512*
* Disturbed consciousness*	*87 (43.9)*	*5 (41.6)*	*0.878*	*0.911*	*0.28-2.97*
* Septic shock*	*30 (15.1)*	*1(8.3)*	*1.0*	*0.509*	*0.063-4.09*
* Sensory disturbance*	*29 (14.6)*	*0*	*0.379*	*0.934*	*0.898-0.971*
* Hemiparesis*	*95 (47.9)*	*4 (33.3)*	*0.324*	*0.542*	*0.158-1.859*
* Visual disturbance*	*27 (13.6)*	*1 (8.3)*	*1.0*	*0.576*	*0.071-4.641*
* Neck Stiff neck*	*59 (29.8)*	*5 (41.6)*	*0.519*	*1.683*	*0.513-5.517*
* Facial palsy*	*12 (6)*	*1 (8.3)*	*0.545*	*1.409*	*0.168-11.842*
* Seizure*	*50 (25.2)*	*0*	*0.074*	*0.925*	*0.885-0.967*
* Speech disturbance*	*8 (4)*	*0*	*1.0*	*0.941*	*0.909-0.974*
* concomitant bacterial meningitis*	*74 (37.4)*	*4 (33.3)*	*1.0*	*0.838*	*0.244-2.879*
*Treatment*					
* Combination of Surgical and antimicrobial therapy*	*162 (81.8)*	*10 (83.3)*	*1.0*	*1.11*	*0.23-5.29*
* Antimicrobial treatment only*	*36 (18.2)*	*2 (16.6)*			
*Outcome*					
* Death*	*41 (20.7)*	*2 (16.7)*	*1.0*	*0.766*	*1.61-3.63*
* Median (IRQ) hospitalization days*	*44 (28–66.5)*	*35 (25.5- 56.8)*	*0.501*		
* Median (IQR) GOS at discharge*	*4 (2–5)*	*4 (1.3 5.0)*	*0.544*		
* Median (IQR) GOS at a minimum of 18 month follow-up*	*5 (5–5)*	*5 (4.3- 5.0)*	*0.385*		

*Abbreviations: IVROBA, Intra-ventricular rupture of brain abscess; GOS, Glasgow Outcome Scale; IQR, inter-quartile range; GCS, Glasgow Coma Scale; β, neuro-imaging findings upon presentation*.

### Causative pathogens and neuro-imaging findings

The causative pathogens between patients with or without NPC were listed in Table 2. Gram-negative bacilli were the most common causative pathogens, followed by *Streptococcus* species, anaerobic pathogens, and *Staphylococcus* species in non-NPC patients, while viridans streptococci and *Staphylococcus aureus* were the two common causative pathogens in the NPC groups. The interval between onset of symptoms to detection of brain abscess between the NPC and non-NPC groups were 10.9 and 13.4 days (*p* = 0.674), respectively. The portal of entry for infection in the 12 NPC cases included hematogenous spread from remote foci (e.g. pulmonary origin) in one, contiguous infection from the para-meningeal foci (e.g. otogenic origin) in three, both post-neurosurgical states and contiguous infection from the para-meningeal foci in six, and unknown in two.

**Table 2:** Causative pathogens of bacterial brain abscesses without or without NPC

	***Non-NPC patients***	***NPC patients***
	***n = 198 (%)***	***n = 12 (%)***
*Gram-negative bacilli (n = 50)*	*49 (24.7)*	*1*^*α*^*(8.3)*
*Streptococcus species (n = 43)*		
* Viridans streptococci*	*31 (15.7)*	*4 (33.3)*
* Other Streptococci*	*8 (4.0)*	*0*
*Anaerobic pathogens (n = 38 )*	*37 (18.7)*	*1*^*β*^*(8.3)*
*Staphylococcus species (n = 21)*		
* Staphylococcus aureus*	*9 (4.5)*	*2 (16.7)*
* Other Staphylococcus species*	*10 (5.1)*	*0*
*Mixed bacterial pathogens (n = 19)*	*18 (9.1)*	*1*^*γ*^*(8.3)*
*Negative culture (n = 39)*	*36 (18.2)*	*3 (25)*

*Note: α, Pseudomonas aeruginosa; β, Fusobacterum nucleatum; γ,Staphylococcus aureus, Enterococcus species, Pseudomonas aeruginosa*.

**Figure 1:**
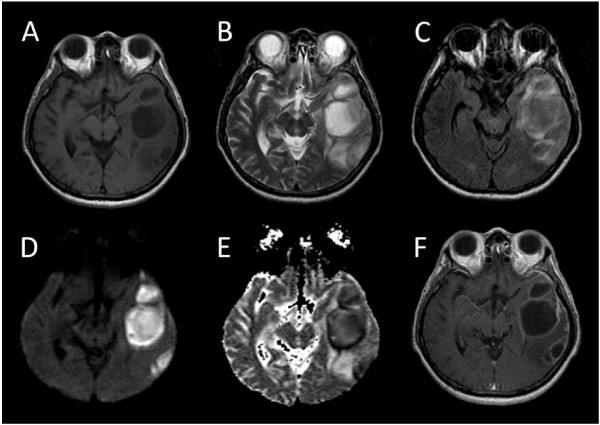
**(A) Pre-contrast axial T1-weighted MR revealed multiple left temporal lesions with hypo-intense central cavity and moderately hypo-intense surrounding edema.(B)** T2-weighted and **(C)** fluid attenuated inversion recovery (FLAIR) axial MR revealed multiple hyper-intense lesions and surrounding hyper-intense edema. **(D)** Diffusion-weighted MR demonstrated high signal intensity, with **(E)** a corresponding reduction in the apparent diffusion coefficient. This was directly related to the cellularity and viscosity of the pus contained within the abscess cavities. In contrast, tumors with central necrosis had an appearance of marked hypo-intensity on diffusion-weighted images and hyper-intensity on the apparent coefficient map. **(F)** Post-gadolinium T1-weighted scan showed thick ring enhancement and increased enhancement of the adjacent dura

The locations of bacterial brain abscesses in the two patient groups were listed in Table 3. The most common sites were the fronto-parietal lobe, followed by the parieto-occipital lobe and the temporal lobe in the non-NPC group. In the NPC group, the temporal lobe was the most common site. Other neuro-imaging findings, including hydrocephalus, rupture into the ventricle, multi-loculated character, and the median (inter-quartile range [IQR]) volume of bacterial brain abscesses on admission was also listed in Table 3 and Figure 1.

**Table 3:** Neuro-imaging findings

	***Non-NPC patients***	***NPC patients***
	***n = 198 (%)***	***n = 12 (%)***
*Location*		
* Single site*		
* Frontal lobe*	*15 (7.5)*	*0*
* Fronto-parietal area*	*23 (11.6)*	*1 (8.3)*
* Fronto-parito-temporal area*	*17 (8.6)*	*1 (8.3)*
* Basal ganglion*	*12 (6.1)*	*0*
* Temporal lobe*	*17 (8.6)*	*6 (50)*
* Parieto-occipital area*	*22 (11.1)*	*1 (8.3)*
* Parietal lobe*	*15 (7.6)*	*0*
* Temporo-parietal area*	*16 (8.0)*	*0*
* Thalamus*	*4 (2.0)*	*0*
* Occipital lobe*	*3 (1.5)*	*0*
* Cerebellum*	*9 (4.5)*	*1 (8.3)*
* Multiple sites*	*45 (22.7)*	*2*^*δ*^*(16.6)*
*Multiloculated*	*41 (20.7)*	*3 (25)*
*Rupture into ventricle*	*62 (31.3)*	*5 (41.7)*
*Hydrocephalus*	*32 (16.2)*	*3 (25)*
*Median (IRQ) volumes of brain abscess (cm*^*3*^*)*^*β*^	*13.19 (4.03-25.13)*	*17.49 (7.47-28.162)*

Note: β, neuro-imaging findings upon presentation; δ, located on the left temporal lobe; IQR, inter-quartile range.

### Clinical characteristics and outcome of patients

Treatment of the bacterial brain abscesses between two patient groups was listed in Table 1. In total, 167 of 210 patients survived. The fatality rate in patients with and without NPC was 16.7% (2/12) and 20.7% (41/198), respectively. The median GOS (inter-quartile range [IQR]) upon discharge between the NPC and non-NPC patients were 4 (1.3-5.0) and 4 (2–5), respectively (*p* = 0.544). The median GOS (IQR) after a minimum 18-month follow-up between the patients with and those without NPC were 5 (4.3-5.0) and 5, respectively (*p* = 0.383). The median (IQR) hospitalization days were 35 (25.5- 56.8) and 44 (28–66.5), respectively (*p* = 0.501).

Antimicrobial therapy with or without surgical intervention (aspiration or total excision) was the cornerstone of treatment in the 210 patients. Forty-eight patients received antimicrobial therapy alone while the other 162 received both antimicrobial therapy and surgical intervention. Among the latter 162 cases, 113 underwent excision of the brain abscesses and 49 underwent aspiration procedures. The fatality rate in patients with excision and aspiration was 22.1% (25/113) and 20.4% (10/49), respectively (*p* = 0.849) (Table 1).

### Clinical comparisons between NPC and non-NPC patients

Comparisons of clinical features and neuro-imaging findings between NPC and non-NPC patients after a minimum 18-months follow-up were listed in Table 1. Statistical analysis between the two patient groups revealed that chronic otitis media (*p <* 0.0001) as the underlying disease, neuro-imaging findings of post-radiation necrosis (*p* < 0.0001), and presence of a temporo-parietal distribution of bacterial brain abscesses (*p* = 0.026) were significant variables. However, the presence of the temporal lobe as the most common site of brain abscesses was not considered significant by Bonferroni’s correction.

Variables used in the stepwise logistic regression model included chronic otitis media as the underlying diseases and neuro-imaging findings of post-radiation necrosis. After analysis, only chronic otitis media as the underlying diseases (*p* = 0.001, OR = 11.06, 95% CI: 2.75-44.58) was independently associated with the NPC group.

## Discussion

Although the incidence of NPC is far higher in Taiwan [1], there is limited data on the global incidence of NPC in patients with bacterial brain abscess. The clinical characteristics of bacterial brain abscess in adult NPC patients is rarely reported in literature [2-8], whereas its frequency in NPC following radiotherapy vary with case determination and inclusion criteria, time period, geographic distribution, and age [2,3,11]. One study on brain abscesses conducted in Hong Kong during the period January 1999 to June 2008 demonstrated that 33% of patients had previous radiotherapy for NPC [15]. There was also a trend towards higher in-patient mortality in patients with NPC-related brain abscess. Another study of brain abscesses conducted in South Africa covering a 20-year period (1983–2002) revealed that none of the 973 patients had NPC as the underlying disease [16].

In Taiwan, the frequency in reported studies varies from an estimated 1% to 4% [2,3]. The incidence rate of bacterial brain abscesses in NPC patients following radiotherapy is around 0.5% in an unpublished data. In the current hospital-based study, NPC following radiotherapy as the underlying diseases occurs in 12 out of 210 patients with bacterial brain abscesses (5.7%). The fatality rate in patients with NPC and in those without is 16.7% and 20.7%, respectively. Except for the temporal lobe as the most common site of brain abscesses being common in NPC-related brain abscess, the causative pathogens and clinical features are similar among these studies.

### Study major findings

The present study makes a comparison between bacterial brain abscess patients with underlying NPC and those without, and produced three major findings. First, chronic otitis media as the underlying disease and the temporal lobe as the most common site of brain abscesses are significantly associated with NPC patients following radiotherapy. Patients with NPC frequently develop otitis media with effusion and that the obstruction and destruction of the Eustachian tube by either tumor or radiotherapy causes immune or mucosal changes in the nasopharynx and middle ear [17]. Further, the risk of CNS infection is increased after radiotherapy with impaired CNS barrier by high prevalence of chronic otitis media. This may explain why most of these cases have the temporal lobe as the most common site of brain abscesses. Furthermore, the concomitant neurosurgical procedures have made these patients more prone to developing brain abscess. Second, there is a high incidence of concomitant with post-radiation necrosis (25%, 3/12) in the NPC patients following radiotherapy. One of the major post-radiation therapy complications is necrosis of otherwise normal surrounding soft tissues and/or bone [18]. The reported incidence of late radiation necrosis after radiotherapy for NPC is 1% [18]. The latent interval between the end of the dose and the onset of symptoms ranges from 9 months to 16 years. This condition is under-diagnosed because many patients have vague symptoms or are asymptomatic [18]. Establishing the diagnosis of brain abscess is not always a straightforward process and radiation necrosis can mimic brain abscess or brain tumor on neuro-imaging, thereby posing significant diagnostic challenges [19]. The rational use of new diagnostic techniques like diffusion-weighted MRI, perfusion-weighted MRI, and magnetic resonance spectroscopy are useful for differentiating between brain abscess and brain tumor or post-radiation necrosis [20]. Imaging study alone cannot differentiate brain abscessed caused by variable pathogens. Thus, pathologic confirmation and/or microbiologic studies are needed to ensure proper management. Third, streptococcus and staphylococcus species are the major causative pathogens in this select group of patients, although Gram-negative bacilli are the most frequently implicated pathogens in bacterial brain abscesses in Taiwan [3].

### Study limitations

There are five main limitations to this study. First, this is a retrospective analysis and is therefore subject to bias of unmeasured factors (e.g. possible reporting bias due to several patients with altered consciousness). Second, only patients with focal neurologic signs at the ER underwent brain CT studies in the study institution. The findings may underestimate the “true” frequency of bacterial brain abscess in NPC patients. Third, the study hospital provides both primary and tertiary referral care for patients, and several patients who were initially treated in other hospitals but subsequently transferred to the hospital for further therapy were also included in this study. It is possible that there is reporting bias due to patient selection. Fourth, despite the small sample of NPC cases (5.7%, 12/210) in this study, the numbers of variables considered for multiple logistic regression analysis was small after Bonferroni correction of the bi-variate analysis. Only associations with *p* < 0.001 was considered significant. Based on the stepwise procedures, only one variable was selected as the important variable predicting outcome. As such, the maximum likelihood estimates of the coefficients are valid in the analysis. Lastly, the choice of therapeutic strategy for the bacterial brain abscesses was different for each patient according to the preference of his/her doctor. This may have caused a potential bias in the statistical analysis.

The portals of entry for infection due to hematogenous spread from remote foci or concomitant bacterial meningitis have a more fulminant course, with higher prevalence of disturbed consciousness, bacteremia, seizure and shock [11]. The mode of entry for infection in the NPC patients is contiguous infection from para-meningeal foci with or without post-neurosurgical states. This implies that the therapeutic outcome cannot be inferior to those of the non-NPC patient group in this study. Clinical features are also similar between two patient groups.

## Conclusions

In summary, NPC patients with bacterial brain abscess frequently have COM as the underlying disease. Neuro-imaging findings often reveal both post-radiation necrosis and the temporal lobe as the most common site of brain abscesses. Establishing the diagnosis of brain abscess is not always a straightforward process and radiation necrosis can mimic brain abscess on neuro-imaging, thereby posing significant diagnostic challenges. Early diagnosis and treatment is essential for survival.

## Competing interests

The authors indicated no potential conflicts of interest.

## Authors’ contribution

PHF had substantial contributions to the conception and design, data acquisition and analysis, and drafting and revision of the manuscript. HWC had substantial contributions to statistical analysis. WCL, NWT, WNC, CRH, TLH, HCL, YJL, BCC, YJ S, and CTK had substantial contributions to the conception and design, and clinical data analysis. CHL and HCW had substantial contributions to conception and design, data analysis, critical revision, and final approval of the revision. “All authors have read and approved the final manuscript.”

## Ethics approval

The study was approved by Chang Gung Memorial Hospital’s Institutional Review Committee on Human Research.

## Pre-publication history

The pre-publication history for this paper can be accessed here:

http://www.biomedcentral.com/1471-2334/12/204/prepub
